# Low Expression of the Polycomb Protein RING1 Predicts Poor Prognosis in Human Breast Cancer

**DOI:** 10.3389/fonc.2020.618768

**Published:** 2021-02-09

**Authors:** Song Gao, Si-Yu Wang, Xing-Da Zhang, Hao Wu, Da Pang

**Affiliations:** ^1^ Department of Breast Surgery, Harbin Medical University Cancer Hospital, Harbin, China; ^2^ Translational Medicine Research and Cooperation Center of Northern China, Heilongjiang Academy of Medical Sciences, Harbin, China; ^3^ Genomics Research Center, State-Province Key Laboratories of Biomedicine-Pharmaceutics of China, College of Pharmacy, Harbin Medical University, Harbin, China; ^4^ Sino-Russian Medical Research Center, Harbin Medical University Cancer Hospital, Harbin, China; ^5^ Heilongjiang Academy of Medical Science, Harbin Medical University, Harbin, China

**Keywords:** ring finger protein1 (RING1), breast cancer, prognostic factor, differentially expressed genes, survival, biomarker, functional enrichment analysis

## Abstract

**Background:**

To date, breast cancer remains the most common malignant tumor in women. In recent years, a growing number of studies on polycomb proteins have been conducted. The Ring finger protein1 (RING1), an essential component of the polycomb family of proteins, plays vital roles in the tumorigenesis of various cancer types. However, further research is required in determining RING1 expression and prognostic value in breast cancer.

**Method:**

RING1 expression level in multiple cancer types was evaluated using the XENA and UALCAN databases. Real-time quantitative PCR (real-time qPCR) and immunohistochemistry (IHC) were used to confirm this expression. The prognostic value was analyzed using our follow-up data and the Kaplan–Meier plotter website. RING1 co-expressed genes and its promoter methylation level were calculated using the cBioPortal and UALCAN online tools. The gene ontology (GO) and the Kyoto encyclopedia of Genes and Genomes (KEGG) pathway enrichment were analyzed using the DAVID online analysis tool.

**Result:**

RING1 expression was upregulated in CHOL (Bile Duct Cancer), ESCA (Esophageal Cancer), LIHC (Liver Cancer), and PCPG (Pheochromocytoma & Paraganglioma). However, its expression level was decreased in COAD (Colon Cancer), KICH (Kidney Chromophobe), KIRP (kidney papillary cell carcinoma), THCA (Thyroid Cancer), and BRCA (Breast carcinoma). RING1 low expression is an unfavorable prognostic factor in many cancer patients, especially in breast cancer patients. For breast cancer, the IHC result showed that RING1 protein expression significantly and negatively correlates with tumor size (*P* = 0.029), LNM (*P* = 0.017), TNM stage (*P* = 0.016), ER (*P* = 0.005), Ki67 (*P* = 0.015), and p53 status (*P* = 0.034). Moreover, the multivariate Cox regression model indicated that RING1 (*P* = 0.038) and ER (*P* = 0.029) expressions were independent prognostic markers for breast cancer. RING1 co-expressed genes were selected and included HDAC10, PIN1, CDK3, BAX, and BAD. GO analysis and KEGG pathway analyses revealed that RING1 related genes, were mainly enriched in “regulation of transcription”, “apoptotic process”, “protein transport”, “protein binding”, “Notch signaling pathway”, and “Homologous recombination”.

**Conclusion:**

RING1 expression was downregulated in breast cancer, and its low expression was associated with worse disease outcomes. RING1 may act as a new prognostic biomarker for breast cancer.

## Introduction

The polycomb group of proteins consists of two major polycomb repressive complexes (PRCs): PRC1 and PRC2 (1) that are known to play an important role in stem cells’ self-renewal (2) and in the regulation of tumorigenesis (3, 4). The transcription factor RING1 is a member of PRC1 that contains the RING finger motif. It has been found to be associated with several regulatory proteins and plays an important role in regulating cell proliferation and transformation (5–7). Recent studies have showed that RING1 is overexpressed in various types of human cancers, including lymphoma, non-small cell lung cancer, and prostate and liver cancers (8–12). RING1 interacts with several human PcG proteins, indicating that RING1 plays an important role within the PcR complex. RING1 overexpression leads to cellular transformation *via* enhancing the expression of the proto-oncogenes, c-jun, and c-fos (5). A recent study reported that Ring1 is upregulated in hepatocellular carcinoma tissues and that it can directly ubiquitinates p53 and promotes cancer cell proliferation, leading to poor outcomes in patients (11). Moreover, RING1 overexpression induces the transformation of hepatic progenitor cells into cancer stem cells through the activation of the Wnt/*β*-catenin signaling pathway (13). These pieces of evidence highlighted the importance of RING1 in malignant transformation and hepatocellular carcinoma development. However, RING1 role in breast cancer is largely unexplored.

In this study, the dysregulation of RING1 was observed in multiple types of cancers and was associated with poor outcomes. Moreover, we found that the low RING1 expression predicts poor survival in breast cancer. We further investigated the relationship between RING1 expression and breast cancer clinicopathological features. Then the potential function of RING1 was explored using bioinformatic analyses of public datasets. Overall, the obtained results indicate that RING1 is associated with breast cancer tumorigenesis and that it can be used as a prognostic biomarker for breast cancer.

## Method and Materials

### The Ring Finger Protein1 mRNA Expression, Methylation Status, and Survival Analysis

The UCSC XENA browser (14) was used for the evaluation of RING1 mRNA expression level, methylation status, and overall survival time in breast cancer and Pan-cancer. UALCAN (http://ualcan.path.uab.edu) is a comprehensive, user-friendly, and interactive web resource for analyzing TCGA and MET500 data, and that was used to explore the mRNA expression and methylation status based on various molecular subtypes of breast cancer (15). The Kaplan–Meier plotter (http://kmplot.com//analysis) can assess the prognostic value of 54k genes (mRNA, miRNA, protein) in 21 cancer types. The primary aim of this tool was to discover and validate survival biomarkers that were mainly based on GEO, TCGA, and EGA databases (16). In this study, Kaplan–Meier plotter in breast cancer or Pan-cancer was used to detect the OS, RFS, and DMFS of RING1 to explore its prognostic value in human cancers.

### Tissue Microarrays and Immunohistochemical Staining

A tissue microarray (TMA) that included 237 breast cancer samples and 19 normal tissues, and 30 breast cancer and paired normal breast tissue slides that were collected by the Harbin Medical University Cancer Hospital, was generated according to a previously described method (17). Informed consent from each patient who participated in this study was obtained. Briefly, the tissue section was baked, deparaffinized, hydrated, and antigen retrieved in citrate buffer (pH 6.0). The TMA was incubated with a peroxidase blocking solution and then with a RING1 primary antibody at a 1:100 dilution (Abcam, Cambridge, MA, USA). After three times washing with PBS, the slide was incubated with secondary antibodies for 1 h at room temperature, and DAB was used as a chromogen. Subsequently, the section was counterstained with hematoxylin, and the IHC scoring was performed as previously described by Dong et al. (18). RING1 expression was independently analyzed and scored by two observers and was based on the intensity and the distribution of positively stained tumor cells, which were demarcated by nuclear yellow particles. The histologic score was calculated by multiplying the intensity and distribution values. Briefly, the distribution of positive cells was scored as follows: 0 (no positive cells); 1 (<25%); 2 (25–50%); 3 (50.01–75%); and 4 (>75%). The staining intensity was graded as follows: 0 (no signal); 1 (weak); 2 (moderate); and 3 (strong). A total score with a possible range of 0 to 12 was calculated and graded as follows: negative (score: 0), weak (score: 1–2), moderate (score: 3v6) or strong (score: 7–12). Scores of “ negative” and “weak” were considered to indicate low expression levels, whereas scores of “moderate” and “strong’ were considered to indicate high expression levels.

### Screening for The Ring Finger Protein1 Co-Expressed Genes and Correlative Analysis of The Ring Finger Protein1 Expression

cBioPortal (https://www.cbioportal.org/) can analyze the genomic data of tumor samples through multifunctional visualization and realize the integrated analysis of complex tumor genome and clinical features (19). This study used the gene expression information from the BRCA database (TCGA, Nature2012) of the cBioPortal platform and the UALCAN web tool to screen for RING1 co-expression genes. The Spearman correlation coefficient was set to |R|≥0.3 as the screening condition for co-expressed genes. The correlation between RING1 mRNA expression and its promoter methylation level or co-expressed genes was analyzed using cBioPortal.

### Gene Ontology Term and Kyoto Encyclopedia of Genes and Genome Pathway Analysis of The Ring Finger Protein1 Co-Expressed Genes

The Database for Annotation, Visualization, and Integrated Discovery (DAVID; https://david.ncifcrf.gov/) is an integrated biological information database that can provide a comprehensive annotation information on a biological function for a large-scale gene list (20). To understand the function of RING1 co-expression genes, Kyoto Encyclopedia of Genes and Genomes (KEGG) pathway (21) and Gene Ontology (GO) enrichment analyses (22) were analyzed by the DAVID platform.

### Statistical Analysis

The analysis of RING1 expression between two groups was examined by the *t*-test or chi-square using Graphpad prism 8.0 (version 8.0; GraphPad, San Diego, CA, USA). The χ2 test was performed to determine the correlation between RING1 expression and the clinicopathological features using the SPSS software (version 22; SPSS, Chicago, IL, USA). The overall survival was estimated using the Kaplan–Meier method. P < 0.05 was considered significant.

## Results

### The Ring Finger Protein1 Is Differently Expressed in Human Pan-Cancer and Reveals a Unique Prognostic Factor for Cancer Patients

**Figure 1:**
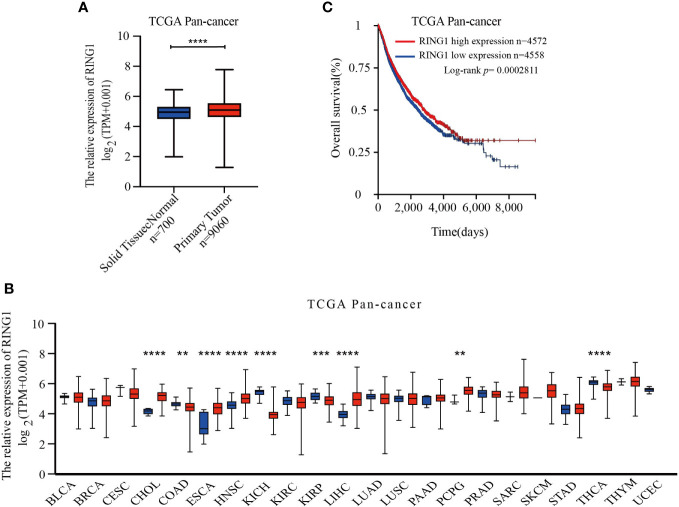
RING1 is differently expressed in human pan-cancer and is a unique prognostic factor for cancer patients. **(A)** RING1 mRNA level was upregulated in primary tumor group compared to normal solid tissues. **(B)** RING1 expression level was analyzed in human pan-cancer according to The Cancer Genome Atlas (TCGA) dataset. The red columns indicate cancer tissues, and the blue columns indicate normal tissues. **(C)** Kaplan–Meier survival analysis for pan-cancer by log-rank test. All the above results were obtained from the XENA website. **p < 0.01; ***p < 0.001; ****p < 0.0001.

RING1 transcriptional level in various human cancers was analyzed using TCGA database. Compared with solid normal tissue groups, RING1 mRNA expression level was significantly upregulated in the primary tumor groups (**Figure 1A**). The result also showed that RING1 expression was upregulated in several types of cancers, including CHOL (Bile Duct Cancer), ESCA (Esophageal Cancer), LIHC (Liver Cancer), and PCPG (Pheochromocytoma & Paraganglioma) (**Figure 1B**) and when compared to their non-cancer counterpart tissues. Besides, we noticed that RING1 expression level was decreased in COAD (Colon Cancer), KICH (Kidney Chromophobe), KIRP (kidney papillary cell carcinoma), and THCA (Thyroid Cancer) when compared to the corresponding normal tissues (**Figure 1B**).

Moreover, RNA-Seq data of 9,130 pan-cancer samples from TCGA datasets was analyzed using the XENA website tools to explore the correlation between the RING1 mRNA expression level and survival status in patients. The Kaplan–Meier curve and log-rank test results revealed that patients with high RING1 mRNA expression level have higher OS (overall survival) compared to the low expression group (**Figure 1C**).

**Figure 2:**
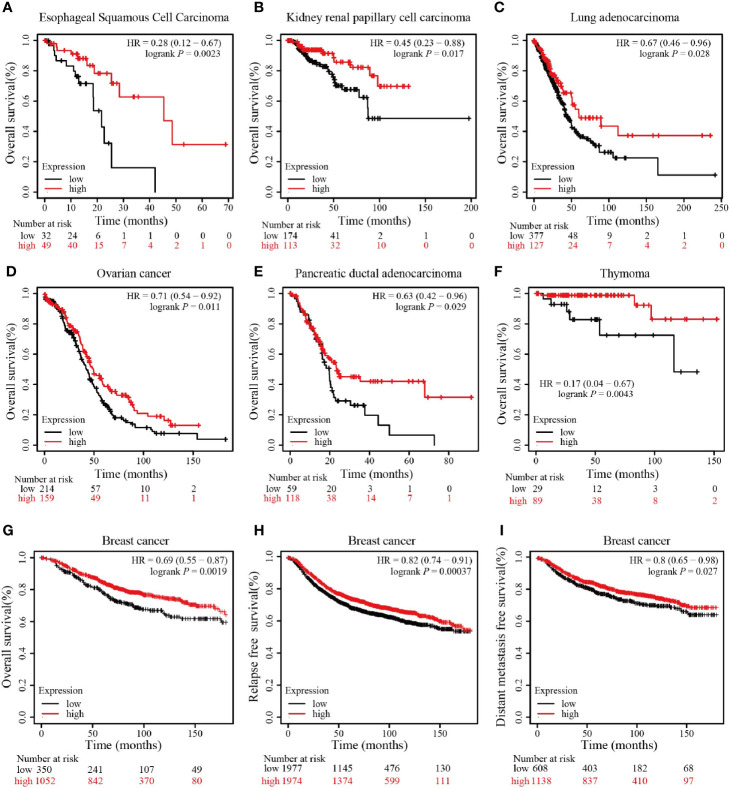
RING1 high expression is associated with better outcome. RING1 high expression is associated with better OS in ESCC **(A)**, KIRP **(B)**, LUAD **(C)**, OV **(D)**, PDAD **(E)**, and THYM **(F)**; RING1 high expression is associated with better OS **(G)**, RFS **(H)**, and DMFS **(I)** in breast cancer. All the above Kaplan–Meier survival curves were performed *via* the Kaplan–Meier plotter web tool. OS, overall survival; RFS, relapse free survival; DMFS, distant metastasis free survival; ESCC, esophageal squamous cell carcinoma; KIRP, kidney papillary cell carcinoma; LUAD, lung adenocarcinoma; OV, ovarian cancer; PDAD, pancreatic ductal adenocarcinoma; THYM, thymoma; HR, hazard ratio.

We further conducted survival analyses using the website of Kaplan–Meier Plotter to determine whether RING1 has a favorable or unfavorable prognostic role in different types of cancers in patients. We found that RING1 low mRNA expression is related to worse OS in ESCC (esophageal squamous cell carcinoma), KIRP (kidney papillary cell carcinoma), LUAD (lung adenocarcinoma), OV (ovarian cancer), PDAD (pancreatic ductal adenocarcinoma), and THYM (thymoma) (**Figures 2A–F**). Breast cancer patients with an increased RING1 mRNA level had better OS, RFS, and DMFS than those with a low expression level (**Figures 2G–I**). When combining these results with the result of the expression profiling analysis, we found that RING1 low expression is an unfavorable cancer prognostic factor.

### Relationship Between The Ring Finger Protein1 Expression and Clinicopathological Parameters of Breast Cancer Patients

**Figure 3:**
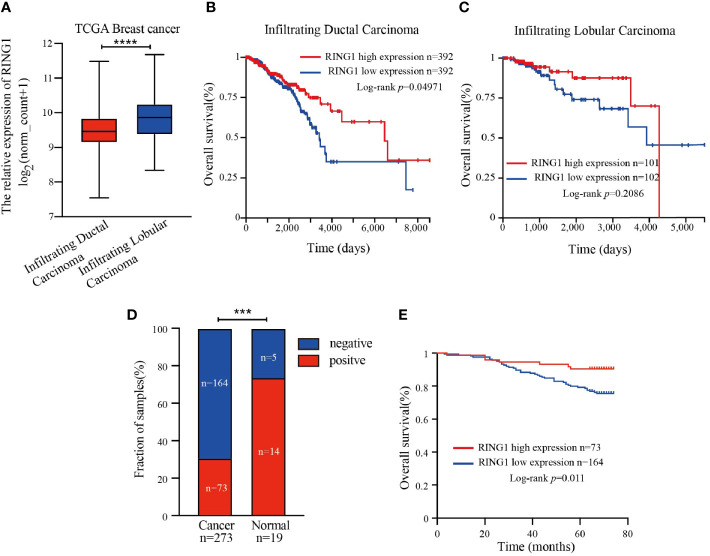
RING1 was downregulated in breast cancer and predicted poor outcome. **(A)** Analysis of RING1 mRNA expression level in IDC and ILC according to the TCGA data. Kaplan–Meier survival analysis for patients with IDC **(B)** or ILC **(C)** by log-rank test and according to the TCGA data. **(A–C)** results were calculated by the XENA web tool. **(D)** Quantification of positive and negative RING1 expression in breast cancer and normal tissues by χ2 test. **(E)** High expression of RING1 is correlated with better overall survival. IDC, Infiltrating Ductal Carcinoma; ILC, Infiltrating Ductal Carcinoma; ***p < 0.001, ****p < 0.0001.

Next, we then focused our study on the role of RING1 in breast cancer. Considering that invasive ductal carcinoma (IDC) and invasive lobular carcinoma (ILC) are the two most common pathological types of breast cancer, we first analyzed RING1 expression level in breast cancer, based on histological subtypes. The results showed that RING1 is downregulated in IDC compared to ILC (**Figure 3A**). Moreover, we found that in the IDC subgroup, patients with a high RING1 expression level had better OS than those with low RING1 expression level (**Figure 3B**). Unfortunately, the relationship between RING1 expression and patient survival status was not found to be statistically significant in the ILC subgroup (**Figure 3C**).

**Figure 4:**
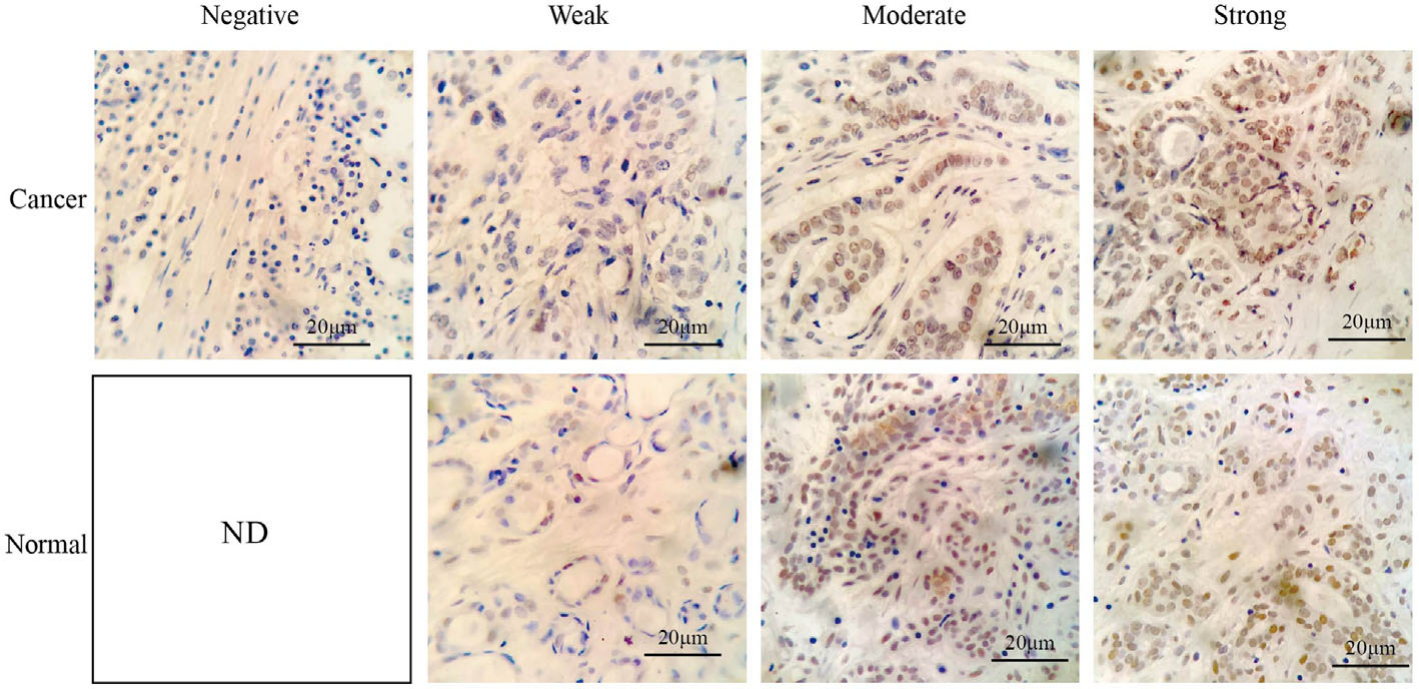
Representative immunohistochemical staining of RING1 for negative, weak, moderate, and strong expression levels in tissue microarray. Scale bar, 20 μm. ND, not detected.

RING1 protein expression in breast cancer and normal tissue was investigated using IHC on a tissue microarray (TMA), containing 237 human breast cancer specimens and 24 normal tissues (**Figure 4**). As shown in **Figure 3D** and **Table 1**, a low RING1 expression level was found in 164 out of 237 (69.2%) breast cancer specimens; however, this level of expression was only found in a small proportion (20.83%) of normal tissues. The results showed a significantly lower RING1 expression in breast cancer compared to that in normal tissues (*P* = 0.0003). Moreover, IHC analysis of 30 breast cancer slides and paired normal tissues also supported the above conclusion (**Supplementary Figure 1**). Consistent with previous research results, we found that RING1 low protein expression was associated with poor overall survival in breast cancer patients (**Figure 3E**). Next, we analyzed the correlation between RING1 protein expression and a series of clinicopathological features, including patient and tumor characteristics. As shown in **Table 2**, RING1 protein expression significantly and negatively correlated with tumor size (*P* = 0.029), LNM (*P* = 0.017), TNM stage (*P* = 0.016), ER (*P* = 0.005), Ki67 (*P* = 0.015), and P53 status (*P* = 0.034). However, there were no significant associations between RING1 protein expression and age (*P* = 0.574), histologic grade (*P* = 0.074), PR (*P* = 0.157), and Her-2 status (*P* = 1.00). Univariate and multivariate analyses were performed to explore the potential clinical significance of RING1 expression in breast cancer. The univariate analysis showed that ER (*P* = 0.008), P53 (*P* = 0.013), and RING1 (*P* = 0.015) were protective factors in breast cancer prognosis, while Her-2 was a risk factor. Moreover, the multivariate Cox regression model indicated that RING1 expression (*P* = 0.038) and ER (*P* = 0.029) were independent prognostic markers (**Table 3**).

**Table 1:** The protein expression level of RING1 in breast cancer compared to normal tissues.

Type	RING1, NO.%		Total	*p*-value
	Positive	Negative		
breast cancer	73(30.80)	164(69.20)	237	<0.0001
normal	19(79.17)	5(20.83)	24	

**Table 2:** Correlation between RING1 expression and clinicopathologic features.

Characteristics	Cases	RING1, NO.%		*p*-value
		Negative	Positive	
Age				
<50	121	86(71.1)	35(28.9)	0.574
≥50	116	78(67.2)	38(32.8)	
Tumor size				
≤2cm	88	53(60.2)	35(39.8)	0.029
>2cm	149	111(74.5)	38(25.5)	
LNM				
<4	173	112(64.7)	61(35.2)	0.017
≥4	64	52(81.2)	12(18.8)	
TNM stage				
I	51	28(54.9)	23(45.1)	0.016
II, III	186	136(73.1)	50(26.9)	
Histologic grade				
I	45	26(57.8)	19(42.2)	0.074
II, III	192	138(71.9)	54(28.1)	
ER status				
Negative	83	67(80.7)	16(19.3)	0.005
Positive	154	97(63.0)	57(37.0)	
PR status				
Negative	47	37(78.7)	10(21.3)	0.157
Positive	190	127(66.8)	63(33.2)	
Her-2 status				
Negative	184	127(69.0)	57(31.0)	1.00
Positive	53	37(64.9)	16(30.2)	
Ki67 status				
≤20%	34	17(50.0)	17(50.0)	0.015
>20%	203	147(72.4)	56(27.6)	
P53 status				
Negative	76	60(78.9)	16(21.1)	0.034
Positive	161	104(64.6)	57(35.4)	

**Table 3:** Prognostic factors in Cox proportional hazards model.

Variables	Univariate analysis			Multivariate analysis		
	HR	95%CI	*P*	HR	95%CI	*P*
ER status						
positive/negative	0.458	0.259–0.812	0.008	0.523	0.293–0.934	0.029
PR status						
positive/negative	0.873	0.434–1.752	0.704			
Her-2 status						
positive/negative	2.006	1.097–3.668	0.024			
Ki67 status						
positive/negative	2.607	0.809v8.396	0.108			
P53 status						
positive/negative	0.484	0.273–0.858	0.013			
RING1 expression						
positive/negative	0.368	0.165–0.823	0.015	0.423	0.187–0.955	0.038

Taken together, these results show that RING1 is expressed a low level in breast tumors and may serve as an unfavorable prognostic maker.

### The Ring Finger Protein1 Expression Is Downregulated by Promoter Hypermethylation

**Figure 5:**
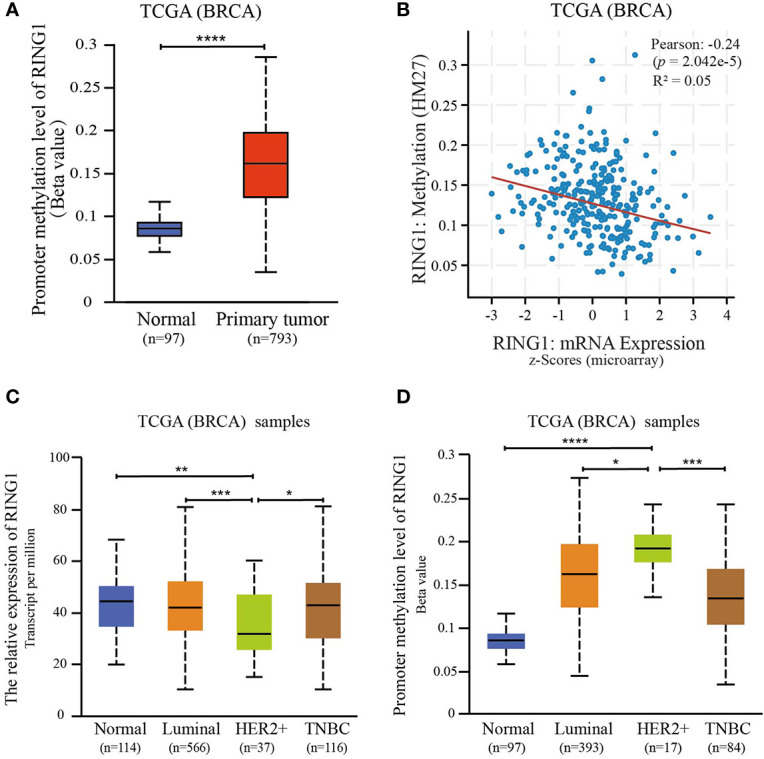
RING1 expression is regulated by promoter methylation. **(A)** Analysis of the levels of RING1 promoter methylation in normal or primary breast tumors according to TCGA using the XENA web tool. **(B)** The correlation between RING1 mRNA expression and its promoter methylation levels was analyzed using the cBioPortal web tool. Analysis of RING1 mRNA expression **(C)** and promoter methylation levels **(D)** according to the PAM50 subgroup using the UALCAN web tool. TCGA, The Cancer Genome Atlas; BRCA, Breast carcinoma; *p < 0.05; **p < 0.01; ***p < 0.001; ****p < 0.0001.

Since promoter hypermethylation is an important cause for a low expression of a tumor suppressor gene in cancer (23, 24), we hypothesized that the low expression of RING1 is regulated by its promoter hypermethylation. To validate this hypothesis, we investigated whether the DNA methylation level of the RING1 promoter was upregulated in breast cancer tissues compared to normal tissues. As expected, using the UALCAN dataset (http://ualcan.path.uab.edu), we found that the level of DNA methylation of the RING1 promoter was higher in breast cancer tissues compared to that in normal tissues (*P* < 0.0001) (**Figure 5A**). Moreover, we also calculated the correlation between RING1 mRNA expression and its promoter DNA methylation *via* the cBioPortal online tool (http://www.cbioportal.org/) for breast invasive carcinoma (The Cancer Genome Atlas, nature 2012), which includes Pearson’s correction. The results showed a significantly negative correlation between RING1 mRNA expression and its promoter DNA methylation level (**Figure 5B**). Furthermore, we analyzed RING1 mRNA expression and its promoter DNA methylation level in each subgroup according to PAM50 subtypes. Compared with the normal, luminal, and TNBC groups, RING1 expression in the HER2 group was significantly reduced; however, its promoter methylation level was significantly increased in the HER2 group (**Figures 5C, D**). Taken together, these data indicate that RING1 expression is reduced due to the upregulation of its promoter hypermethylation.

### Functional and Pathway Enrichment Analyses of The Ring Finger Protein1 Co-Expressed Genes in Breast Cancer

**Figure 6:**
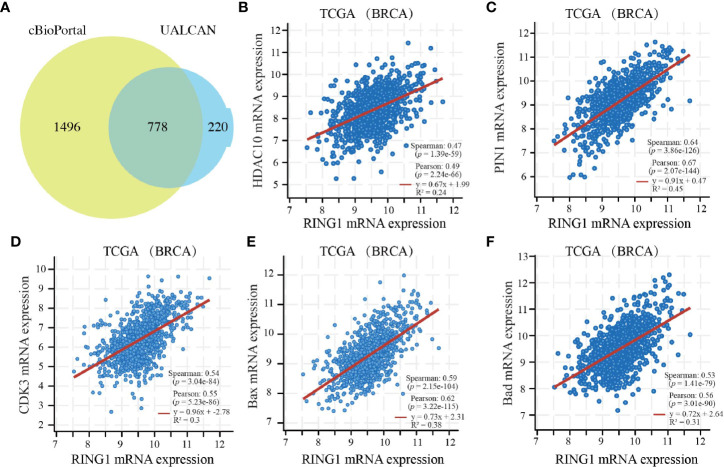
RING1 co-expressed genes in breast cancer. **(A)** A Venn diagram showing RING1 overlapped co-expression genes that were screened by cBioPortal and UALCAN. RING1 mRNA expression correlates with the mRNA expression of HDAC10 **(B)**, PIN1 **(C)**, CDK3 **(D)**, BAX **(E)**, BAD **(F)**. BRCA, breast carcinoma.

**Figure 7:**
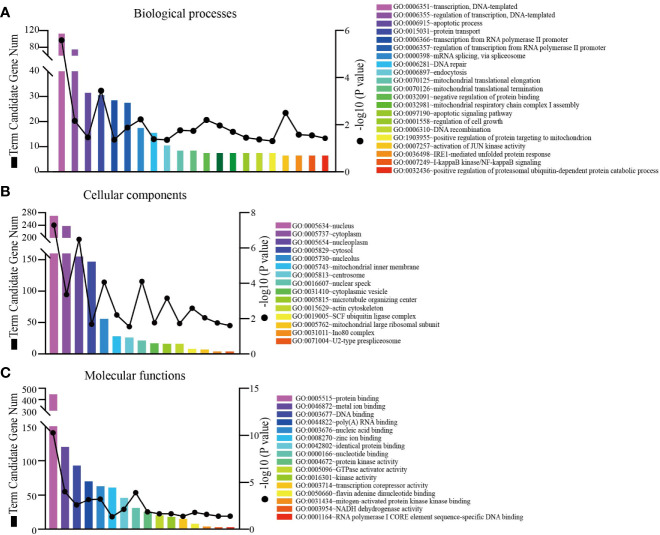
RING1 Go function enrichment and its co-expressed genes in breast cancer was predicted by the DAVID web tool. GO enrichment analysis based on three aspects: **(A)** biological processes, **(B)** cellular components, and **(C)** molecular functions.

**Figure 8:**
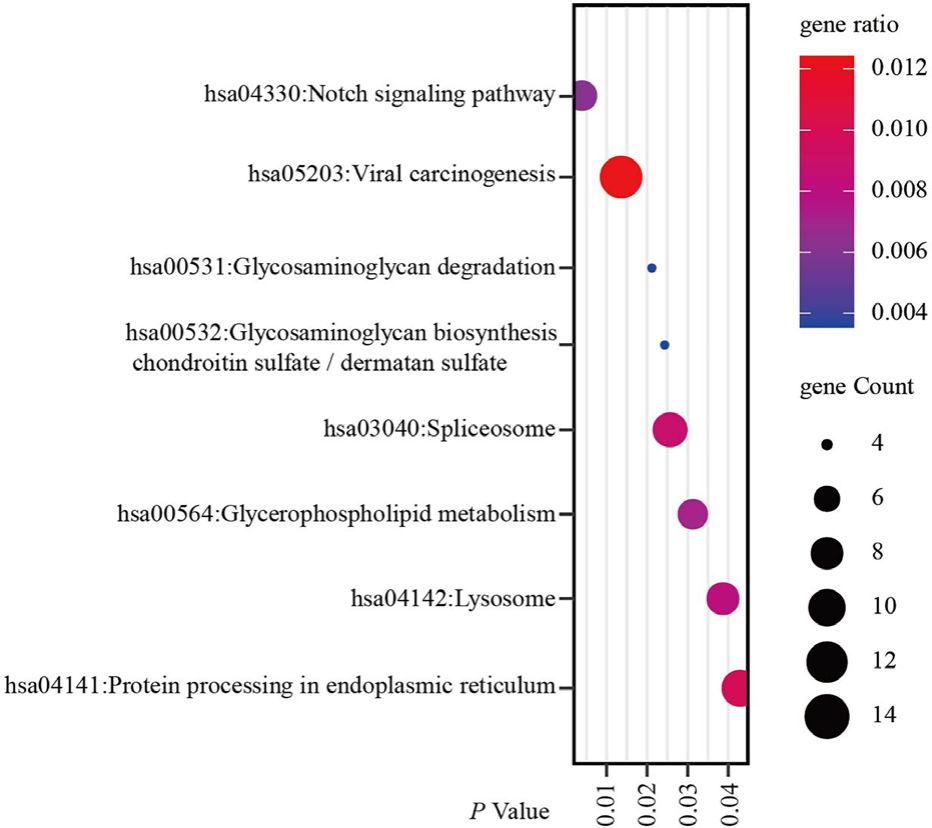
RING1 KEGG pathway enrichment analysis and its co-expressed genes in breast cancer was calculated by the DAVID web tool. KEGG, Kyoto encyclopedia of Genes and Genomes.

Since co-expression gene analysis is a systematic and effective method to analyze the potential regulatory pattern of a target gene (25), RING1 co-expressed genes were calculated by analyzing their mRNA expression *via* the cBioPortal and UALCAN online tools for breast invasive carcinoma (The Cancer Genome Atlas, PanCancer Atlas). A total of 2,275 and 998 co-expressed genes, with a Spearman or Pearson correlation coefficient ≥0.3, were screened through the cBioPortal and UALCAN databases, respectively (**Supplemental File 1**). As shown in the Venn diagram, a total of 778 co-expressed genes overlapped among the two datasets (**Figure 6A**). The results identified several significantly positive co-expressed genes, including HDAC10, PIN1, CDK3, BAX, and BAD (**Figures 6B–F**). Their functional and pathway enrichment analyses were performed using DAVID online tool (https://david.ncifcrf.gov/). The Go enrichment analysis was composed of three aspects: biological processes, cellular components, and molecular functions. For the biological process function, the genes were mainly enriched in GO terms, such as, regulation of transcription, apoptotic process, and protein transport (**Figure 7A**). Notably, the cell component enrichment of those genes mostly involved the nucleus, cytoplasm, and nucleolus (**Figure 7B**). Regarding the molecular function, the genes were mainly enriched in protein, metal ion, and nucleotide binding (**Figure 7C**). The enriched KEGG pathway included the Notch signaling pathway, viral carcinogenesis, spliceosome, homologous recombination, protein processing in the endoplasmic reticulum, and RIG-I-like receptor signaling pathway (**Figure 8**). Taken together, these results suggest that RING1 may play a crucial biological function in breast cancer tumorigenesis.

## Discussion

Due to the limited number of studies, the role of RING1 in breast cancer is unclear. In this study, we found that RING1 is downregulated in breast cancer and that its low level of expression is associated with better OS, RFS and DMFS (**Figure 2** and **Figures 3B–D**). Moreover, we provided evidence that the reduction of RING1 expression is associated with the upregulation of its promoter methylation (**Figure 5**). In light of our results that showed that RING1 protein expression significantly and negatively correlates with tumor size, LNM, TNM stage, ER, Ki67, and P53 status (**Table 2**), it is adequate to suggest that RING1 plays a key role in promoting breast cancer cells’ growth and metastasis, though the activation of p53 protein expression. In addition, the multivariate Cox regression model indicated that RING1 and ER expression are independent prognostic markers (**Table 3**). The bioinformatic analysis indicated that RING1 may be involved in apoptosis and Notch signaling pathway.

Cancer is considered a genetic disease, in which abnormal gene expression causes tumor cells to lose normal characteristics (26). One group of genes that plays a vital role in maintaining normal cellular characteristics belongs to the Polycomb group (PcG). Polycomb group (PcG) proteins consist of two major polycomb repressive complexes: PRC1 and PRC2 (1, 2). RING1 that was first discovered as a transcriptional repressor that exerts tumorigenic activity, is a central component of PRC1. Together with RNF2 and BMI1, these factors act as a E3 ubiquitin ligase that monoubiquitinate histone H2A. However, only a limited number of studies on the role of RING1 in cancer, is available. In this study, we first found that RING1 was abnormally expressed in different type of cancers. RING1 expression was significantly elevated in some cancers, such as liver cancer, whereas its expression was decreased in other solid cancers, such as Colon Cancer (**Figure 1A**). Regarding its prognostic value, previous studies considered that RING1 overexpression is an unfavorable prognostic factor in a part of tumors, however, an obvious heterogeneity that affected the prognostic value, was found in other tumors. These two opposite reports make the exact prognostic value of RING1 uncertain. This study indicated that RING1 overexpression predicts better prognosis in some type of cancers (**Figure 2**).

Previous studies proved that the RING1 protein was ubiquitously expressed in different types of normal tissues. For cancer, recent studies have shown that the abnormal gene expression of RING1 leads to the development of a variety of cancers (27). RING1 was significantly overexpressed in high GS (Gleason score) prostate cancer, extraprostatic extension, and positive surgical margins, and can be used as a valuable predictive marker for PSA recurrence after radical prostatectomy (9). For bladder cancer, a study detected RING1 expression in 85 of 93 samples, but the median relative expression was 19.98 (range 0**–**91.36) (28). Therefore, we speculated that RING1 is expressed at a relatively low level in bladder cancer, which requires further studies to prove. Although an anomalously low expression of RING1 was observed in a few proportions of Hodgkin and Reed-Sternberg (HSR) cells, most of Hodgkin’s lymphoma (HS) cases expressed a high level. Additionally, a RING1 and E2F6 co-expression was found in the same HL cells (10).

RING1 was overexpressed in non-small cell lung cancer (NSCLC), where it promoted cell growth. RING1 high expression significantly correlated with short overall survival and unfavorable prognosis (8). Interestingly, Wang et al. reported that the LncRNA XIST promotes NSCLC growth and metastasis by inhibiting the miR-744/RING1/Wnt/*β*-catenin axis (29). At present, most of the research about RING1 focuses on liver cancer and RING1 overexpression was also observed in hepatocellular carcinoma (HCC) specimens (11–13). RING1 stabilized endogenous p53 protein level by targeting p53 degradation, which ultimately promoted the migration and proliferation of liver cancer cells (11). Chen et al. reported that RYBP (RING1 and YY1 binding protein), another PcG complex member, binds to MDM2 and reduces p53 ubiquitination, leading to its stabilization (30). This result might support the notion that PcG proteins, containing RING1 and RYBP, may be involved in the regulation of p53 expression. RYBP plays a tumor suppressor gene role in breast cancer by inhibiting breast cancer cell proliferation and metastasis (31). Overexpression of RYBP inhibits ESCC proliferation by downregulating CDC6 and CDC45 in the G1-S phase transition and predicted a better outcome of ESCC patients (32). As a binding protein of RING1 and YY1, RYBP can exert a tumor suppressor effect and a good prognostic factor may depend on the role of RING1. These results may indicate that RING1 may play a tumor suppressor genes’ role from another aspect. Zhu et al. found that RING1 overexpression promotes colony formation, cell multiplication and invasion of hepatic progenitor cells (HPCs), and that it also drives their malignant transformation by activating the Wnt/*β*-catenin signal pathway (13). Consistently, Xiong et al. revealed that the upregulation of RING1 expression accelerates the proliferation of HCC cells by promoting cell cycle progression and indicates poor prognosis (12).

To sum up, the expression of RING1 was found to significantly vary among different cancer types suggesting that further research is required to explore the potential role of RING1 cancer. However, recently published studies on RING1 role in cancer are preliminary, and additional *in vivo* and *in vitro* studies are needed to clarify the function of RING1, especially in breast cancer. Importantly, we have provided new evidence that RING1 is a useful biomarker and a prognostic predictor in breast cancer.

## Conclusion

In summary, our study found that RING1 expression was downregulated in breast cancer, and its low expression was associated with worse disease outcomes. RING1 may act as a new prognostic biomarker for breast cancer.

## Data Availability Statement

The original contributions presented in the study are included in the article/**Supplementary Material**; further inquiries can be directed to the corresponding author.

## Ethics Statement

The Medical Ethics Committee of Harbin Medical University Cancer Hospital approved this study, and informed consent was obtained from all the patients.

## Author Contributions

DP, and SG designed the experiments. SG, X-DZ and S-YW carried out the experiments. HW, X-DZ, and SG analyzed the experimental results. SG and HW wrote the manuscript. DP and SG funded the research. All authors contributed to the article and approved the submitted version.

## Funding

This study was supported by the National Natural Science Foundation of China, Song Gao, Grant Numbers: 81902950, Innovation Scientific Research Fund of Harbin Medical University, Song Gao, Grant Numbers: 31041180113, and National Natural Science Foundation of China, Da Pang, Grant Numbers: 81972706.

## Conflict of Interest

The authors declare that the research was conducted in the absence of any commercial or financial relationships that could be construed as a potential conflict of interest.

## Supplementary Material

Supplementary Figure 1
**(A)** Quantification of positive and negative RING1 expression in breast cancer and paired normal tissues by χ2 test. **(B)** Representative immunohistochemical staining of RING1 for negative and positive expression in breast cancer and paired normal tissues. Scale bar, 20 μm.

Supplementary Table 1 Ring1 co-expressed gene list.

## Abbreviations

BAD, BCL2 associated agonist of cell death; BAX, BCL2 associated X; BMI1, BMI1 proto-oncogene; BRCA, Breast carcinoma; CDK3, Cyclin Dependent Kinase 3; CHOL, Bile Duct Cancer; COAD, Colon Cancer; DMFS, Disease metastasis free survival; ER, Estrogen Receptor; ESCA, Esophageal Cancer; ESCC, Esophageal Squamous Cell Carcinoma; GO, Gene Ontology; GS, Gleason score; HCC, Hepatocellular Carcinoma; HDAC10, Histone Deacetylase 10; HR, Hazard Ratio; HS, Hodgkin’s Lymphoma; HSR, Hodgkin and Reed–Sternberg; IDC, Infiltrating Ductal Carcinoma; IHC, Immunohistochemistry; ILC, Infiltrating Ductal Carcinoma; KEGG, Kyoto Encyclopedia of Genes and Genomes; KICH, Kidney Chromophobe; KIRP, Kidney Papillary Cell Carcinoma; KIRP, Kidney papillary cell carcinoma; LIHC, Liver Cancer; LNM, Lymph Node Metastasis; LUAD, Lung adenocarcinoma; NSCLC, Non-Small Cell Lung Cancer; OS, Overall Survival; OV, ovarian cancer; PCPG, Pheochromocytoma & Paraganglioma; PDAD, Pancreatic Ductal Adenocarcinoma; PIN1, peptidylprolyl cis/trans isomerase, NIMA-interacting 1; PR, Progesterone Receptor; PRC, Polycomb Repressive Complexes; PRC1, Polycomb Repressive Complexes 1; PRC2, Polycomb Repressive Complexes 2; RFS, Relapse Free Survival; RING1, Ring finger protein1; RNF2, Ring Finger Protein 2; RYBP, RING1 and YY1 binding protein; TCGA, The Cancer Genome Atlas; THCA, Thyroid Cancer; THYM, Thymoma; TMA, Tissue Microarray; TNM, Tumor Node Metastasis.

## References

[1] Sauvageau M Sauvageau G Polycomb group proteins: multi-faceted regulators of somatic stem cells and cancer. Cell Stem Cell (2010) 7:299–313. 10.1016/j.stem.2010.08.002 20804967 PMC4959883

[2] Aloia L Di Stefano B Di Croce L Polycomb complexes in stem cells and embryonic development. Development (2013) 140:2525–34. 10.1242/dev.091553 23715546

[3] Richly H Aloia L Di Croce L Roles of the Polycomb group proteins in stem cells and cancer. Cell Death Dis (2011) 2:e204–4. 10.1038/cddis.2011.84 PMC3186902 21881606

[4] Bracken AP Helin K Polycomb group proteins: navigators of lineage pathways led astray in cancer. Nat Rev Cancer (2009) 9:773–84. 10.1038/nrc2736 19851313

[5] Satijn DP Otte AP RING1 interacts with multiple Polycomb-group proteins and displays tumorigenic activity. Mol Cell Biol (1999) 19:57–68. 10.1128/mcb.19.1.57 9858531 PMC83865

[6] Satijn DP Gunster MJ van der Vlag J Hamer KM Schul W Alkema MJ RING1 is associated with the polycomb group protein complex and acts as a transcriptional repressor. Mol Cell Biol (1997) 17:4105–13. 10.1128/mcb.17.7.4105 PMC232264 9199346

[7] Satijn DP Olson DJ van der Vlag J Hamer KM Lambrechts C Masselink H Interference with the expression of a novel human polycomb protein, hPc2, results in cellular transformation and apoptosis. Mol Cell Biol (1997) 17:6076–86. 10.1128/mcb.17.10.6076 PMC232457 9315667

[8] Zhou Y Wan C Liu Y Lv L Chen B Ni R Polycomb group oncogene RING1 is over-expressed in non-small cell lung cancer. Pathol Oncol Res (2014) 20:549–56. 10.1007/s12253-013-9727-9 24414991

[9] van Leenders GJLH Dukers D Hessels D van den Kieboom SWM Hulsbergen CA Witjes JA Polycomb-group oncogenes EZH2, BMI1, and RING1 are overexpressed in prostate cancer with adverse pathologic and clinical features. Eur Urol (2007) 52:455–63. 10.1016/j.eururo.2006.11.020 17134822

[10] Sánchez-Beato M Sánchez E García JF Pérez-Rosado A Montoya MC Fraga M Abnormal PcG protein expression in Hodgkin’s lymphoma. Relation with E2F6 and NFkappaB transcription factors. J Pathol (2004) 204:528–37. 10.1002/path.1661 15470680

[11] Shen J Li P Shao X Yang Y Liu X Feng M The E3 Ligase RING1 Targets p53 for Degradation and Promotes Cancer Cell Proliferation and Survival. Cancer Res (2018) 78:359–71. 10.1158/0008-5472.CAN-17-1805 29187402

[12] Xiong Y Hu B Wei L Jiang D Zhu M Upregulated expression of polycomb protein Ring1 contributes to poor prognosis and accelerated proliferation in human hepatocellular carcinoma. Tumour Biol (2015) 36:9579–88. 10.1007/s13277-015-3721-7 26141041

[13] Zhu K Li J Li J Sun J Guo Y Tian H Ring1 promotes the transformation of hepatic progenitor cells into cancer stem cells through the Wnt/β-catenin signaling pathway. J Cell Biochem (2019). 10.1002/jcb.29496 31696964

[14] Goldman MJ Craft B Hastie M Repečka K McDade F Kamath A Visualizing and interpreting cancer genomics data via the Xena platform. Nat Biotechnol (2020) 38:675–8. 10.1038/s41587-020-0546-8 PMC7386072 32444850

[15] Chandrashekar DS Bashel B Balasubramanya SAH Creighton CJ Ponce-Rodriguez I Chakravarthi BVSK UALCAN: A Portal for Facilitating Tumor Subgroup Gene Expression and Survival Analyses. Neoplasia (2017) 19:649–58. 10.1016/j.neo.2017.05.002 PMC5516091 28732212

[16] Lánczky A Nagy Á Bottai G Munkácsy G Szabó A Santarpia L miRpower: a web-tool to validate survival-associated miRNAs utilizing expression data from 2178 breast cancer patients. Breast Cancer Res Treat (2016) 160:439–46. 10.1007/s10549-016-4013-7 27744485

[17] Gao S Ge A Xu S You Z Ning S Zhao Y PSAT1 is regulated by ATF4 and enhances cell proliferation via the GSK3beta/beta-catenin/cyclin D1 signaling pathway in ER-negative breast cancer. J Exp Clin Cancer Res (2017) 36:179. 10.1186/s13046-017-0648-4 29216929 PMC5721480

[18] Dong J Wang R Ren G Li X Wang J Sun Y HMGA2-FOXL2 Axis Regulates Metastases and Epithelial-to-Mesenchymal Transition of Chemoresistant Gastric Cancer. Clin Cancer Res (2017) 23:3461–73. 10.1158/1078-0432.CCR-16-2180 28119367

[19] Cerami E Gao J Dogrusoz U Gross BE Sumer SO Aksoy BA The cBio cancer genomics portal: an open platform for exploring multidimensional cancer genomics data. Cancer Discovery (2012) 2:401–4. 10.1158/2159-8290.CD-12-0095 PMC3956037 22588877

[20] Huang DW Sherman BT Lempicki RA Systematic and integrative analysis of large gene lists using DAVID bioinformatics resources. Nat Protoc (2009) 4:44–57. 10.1038/nprot.2008.211 19131956

[21] Kanehisa M Sato Y Furumichi M Morishima K Tanabe M New approach for understanding genome variations in KEGG. Nucleic Acids Res (2019) 47:D590–5. 10.1093/nar/gky962 PMC6324070 30321428

[22] Makabe T Arai E Hirano T Ito N Fukamachi Y Takahashi Y Genome-wide DNA methylation profile of early-onset endometrial cancer: its correlation with genetic aberrations and comparison with late-onset endometrial cancer. Carcinogenesis (2019) 40:611–23. 10.1093/carcin/bgz046 PMC6610171 30850842

[23] Feinberg AP Tycko B The history of cancer epigenetics. Nat Rev Cancer (2004) 4:143–53. 10.1038/nrc1279 14732866

[24] Esteller M Epigenetics in cancer. N Engl J Med (2008) 358:1148–59. 10.1056/NEJMra072067 18337604

[25] Villa-Vialaneix N Liaubet L Laurent T Cherel P Gamot A SanCristobal M The structure of a gene co-expression network reveals biological functions underlying eQTLs. PloS One (2013) 8:e60045. 10.1371/journal.pone.0060045 23577081 PMC3618335

[26] Spandidos DA Oncogenes and tumor suppressor genes as paradigms in oncogenesis. J BUON (2007) 12 Suppl 1:S9–12. 17935284

[27] Sánchez-Beato M Sánchez E González-Carreró J Morente M Díez A Sánchez-Verde L Variability in the expression of polycomb proteins in different normal and tumoral tissues. A pilot study using tissue microarrays. Mod Pathol (2006) 19:684–94. 10.1038/modpathol.3800577 16528373

[28] Hinz S Kempkensteffen C Christoph F Krause H Schrader M Schostak M Expression parameters of the polycomb group proteins BMI1, SUZ12, RING1 and CBX7 in urothelial carcinoma of the bladder and their prognostic relevance. Tumour Biol (2008) 29:323–9. 10.1159/000170879 18984978

[29] Wang J Cai H Dai Z Wang G Down-regulation of lncRNA XIST inhibits cell proliferation via regulating miR-744/RING1 axis in non-small cell lung cancer. Clin Sci (2019) 133:1567–79. 10.1042/CS20190519 31292221

[30] Chen D Zhang J Li M Rayburn ER Wang H Zhang R RYBP stabilizes p53 by modulating MDM2. EMBO Rep (2008) 10:166–72. 10.1038/embor.2008.231 PMC2637313 19098711

[31] Zhou H Li J Zhang Z Ye R Shao N Cheang T RING1 and YY1 binding protein suppresses breast cancer growth and metastasis. Int J Oncol (2016) 49:2442–52. 10.3892/ijo.2016.3718 27748911

[32] Ke Y Guo W Huang S Li Y Guo Y Liu X RYBP inhibits esophageal squamous cell carcinoma proliferation through downregulating CDC6 and CDC45 in G1-S phase transition process. Life Sci (2020) 250:117578. 10.1016/j.lfs.2020.117578 32209426

