# Supplementary figures and images for: Low Expression of the Polycomb Protein RING1 Predicts Poor Prognosis in Human Breast Cancer
Source: Front Oncol. 2021 Feb 9;10:618768. doi: 10.3389/fonc.2020.618768 (PMC7900562; doi:10.3389/fonc.2020.618768)

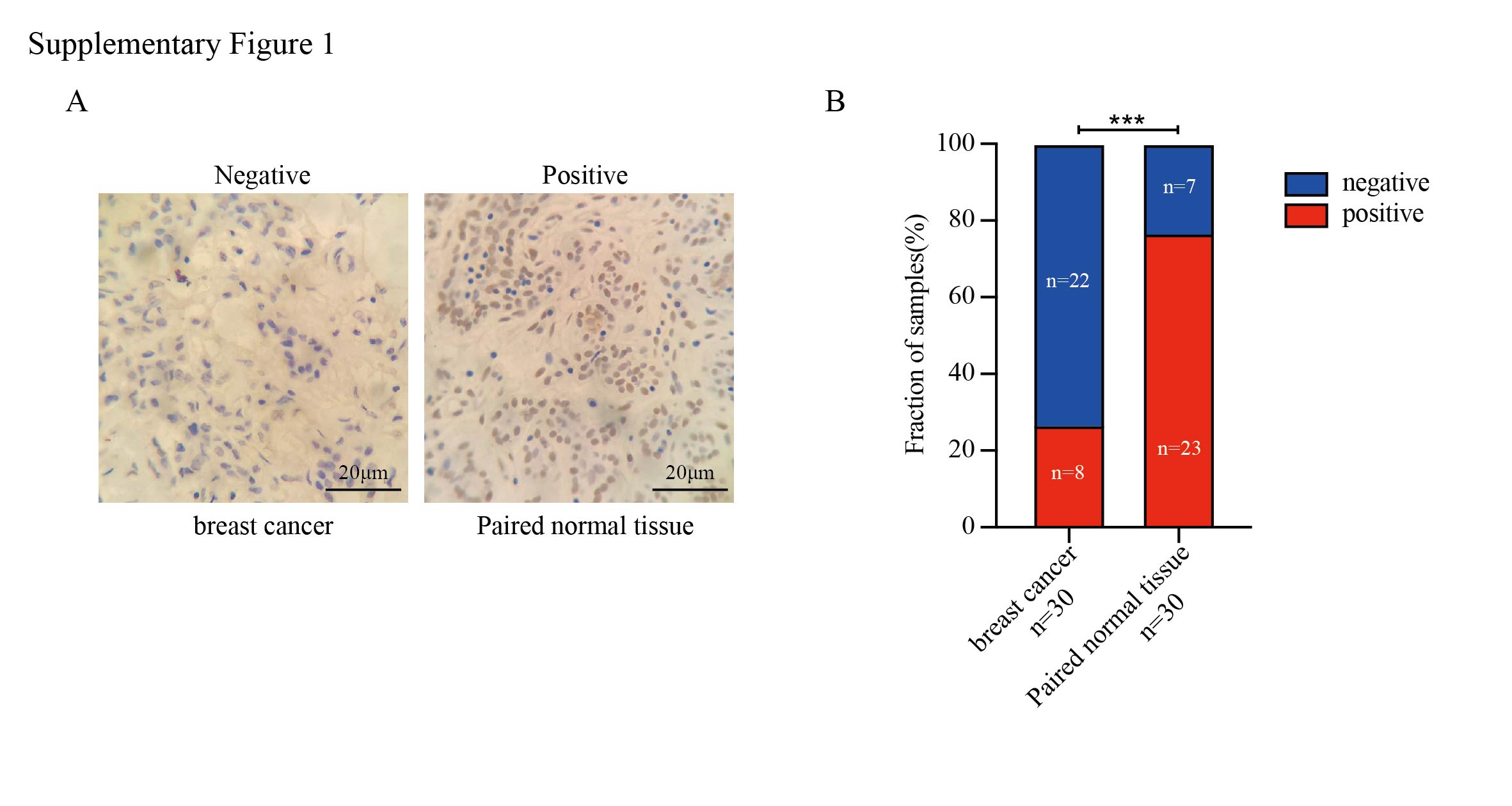

Supplement: Supplementary Figure 1 — (A) Quantification of positive and negative RING1 expression in breast cancer and paired normal tissues by χ2 test. (B) Representative immunohistochemical staining of RING1 for negative and positive expression in breast cancer and paired normal tissues. Scale bar, 20 μm. [file Image_1.jpeg]
